# Res2Net-based multi-scale and multi-attention model for traffic scene image classification

**DOI:** 10.1371/journal.pone.0300017

**Published:** 2024-05-20

**Authors:** Guanghui Gao, Yining Guo, Lumei Zhou, Li Li, Gang Shi

**Affiliations:** School of Computer Science and Technology, Xinjiang University, Urumqi, China; University of Petroleum & Energy Studies, INDIA

## Abstract

With the increasing applications of traffic scene image classification in intelligent transportation systems, there is a growing demand for improved accuracy and robustness in this classification task. However, due to weather conditions, time, lighting variations, and annotation costs, traditional deep learning methods still have limitations in extracting complex traffic scene features and achieving higher recognition accuracy. The previous classification methods for traffic scene images had gaps in multi-scale feature extraction and the combination of frequency domain, spatial, and channel attention. To address these issues, this paper proposes a multi-scale and multi-attention model based on Res2Net. Our proposed framework introduces an Adaptive Feature Refinement Pyramid Module (AFRPM) to enhance multi-scale feature extraction, thus improving the accuracy of traffic scene image classification. Additionally, we integrate frequency domain and spatial-channel attention mechanisms to develop recognition capabilities for complex backgrounds, objects of different scales, and local details in traffic scene images. The paper conducts the task of classifying traffic scene images using the Traffic-Net dataset. The experimental results demonstrate that our model achieves an accuracy of 96.88% on this dataset, which is an improvement of approximately 2% compared to the baseline Res2Net network. Furthermore, we validate the effectiveness of the proposed modules through ablation experiments.

## Introduction

With rapid urbanization and increasing transportation modes, urban transportation problems have become inevitable in people’s daily lives. However, these problems, including traffic congestion and accidents, pose significant challenges to urban transportation systems. Such congestion in urban areas results in travel delays, increased fuel consumption, and a higher risk of accidents. Moreover, it significantly impacts travel safety and hinders urban development. Hence, effective monitoring and detecting of traffic conditions are crucial for efficient road traffic management. In addition to congestion, rapid traffic accident detection is paramount, given the staggering global statistics. Approximately 1.35 million people annually lose their lives in road accidents worldwide, making road traffic injuries the eighth leading cause of death across all age groups and the leading cause of death for individuals aged 5-29 [1]. However, people often overlook one crucial metric related to post-accident survival—the time between an accident and the dispatch of emergency medical personnel to the scene. Reducing this response time can decrease mortality rates by 6% [2]. Many countries and regions have implemented traffic signals and surveillance cameras to mitigate traffic accidents and their consequences. However, these measures often require substantial human and material resources and may not detect accidents in real time efficiently. The combination of intelligent transportation and artificial intelligence is currently a research hotspot [3], presents a hybrid feature selection-based machine learning classification approach for detecting significant attributes and predicting injury severity in single and multiple-vehicle accidents [4], proposes an innovative two-step method and formulate a support vector regression (SVR) model into the state-space model (SSM) framework for traffic crash prediction.

In addition to machine learning, traffic scene image classification technology is also an important research direction. The significance of this study lies in improving the neural network model for tasks of traffic scene image classification, enhancing classification accuracy, advancing the development of algorithms in this domain, obtaining more precise classification results, improving the accuracy of traffic scene understanding and analysis, and contributing to the enhancement of applications in traffic management, traffic safety, and traffic decision-making. A more accurate model for traffic scene image classification is valuable for traffic management departments in acquiring essential information about the traffic environment, providing robust support for traffic management. Furthermore, the technology of traffic scene image classification holds extensive potential in fields such as urban planning, traffic safety management, and intelligent transportation systems. Image classification of traffic scenes falls under the domain of scene recognition and classification, which is a significant sub-field in computer vision. Its objective is to assign input images of various scenes to predefined categories for improved understanding and analysis of objects and behaviors within those scenes. While humans rely on deep comprehension of images to form high-level abstract concepts for scene recognition, computers primarily rely on the digital storage format of images to make judgments. This discrepancy between the conceptual similarity understood by humans and the similarity derived from the digital storage format understood by computers is known as the Semantic Gap, representing a significant challenge in image scene recognition. Bridging this Semantic Gap is crucial for enhancing the accuracy and effectiveness of image scene recognition systems. Traditional machine-learning approaches have primarily focused on feature extraction and classifier design. Early studies often utilized manually designed feature extractors such as HOG [5] and SIFT [6], followed by traditional classifiers like SVM and naive Bayes for image or video classification. Although these methods achieved specific results, the adaptability of handcrafted features to different scenes and tasks could have been improved, particularly in complex scenes.

In contrast, through end-to-end training, deep learning methods can automatically learn feature representations and classifiers, eliminating the limitations associated with handcrafted features in traditional methods. The development of feature extraction networks and image classification mutually reinforce each other. Feature extraction networks aim to enhance features’ expressive power and efficiency, resulting in significant improvements in image classification performance. Conversely, the requirements of image classification tasks drive the development of feature extraction networks, spurring researchers to propose new networks to accommodate diverse datasets and tasks. Convolutional neural networks (CNNs) are the most widely used models among deep learning-based methods. The adaptive feature representation learning capability of CNNs enables more accurate classification than traditional methods. Currently, CNN-based approaches have become mainstream in traffic scene recognition and classification, with an increasing number of CNN-based network models emerging.

This paper proposes a series of enhancements to the res2net network. We introduce the Adaptive Feature Refinement Pyramid module, which improves the network’s ability to fuse multi-scale features, thereby capturing more comprehensive feature information. We also introduce the Frequency Domain Attention and Spatial-Channel and Similarity Attention modules to provide a more holistic feature representation, enhancing model performance and interpretability. The main contributions of this work are as follows:

(1) This paper proposes the Adaptive Feature Refinement Pyramid Module (AFRPM) by improving the FPN structure [7] and incorporating the SE module [8], aiming to enhance multi-scale feature fusion.
(2) This paper also proposes the frequency-domain attention module, FDAM, and the SCSAM module, which is a fusion of the spatial channel and similarity attention module [9], to enhance the performance of the model in terms of the four aspects of the attention mechanism: frequency-domain, spatial, channel, and neuron.
(3) Compared to traditional deep learning methods or baseline models, we have achieved a significant improvement in classification accuracy on the Traffic-Net dataset [10]. Our approach enables more accurate identification of various scenarios and variations in traffic scene images, providing new insights and practical methods for research in the field of traffic scene image classification.

The structure of the paper is as follows: The Related Works section provides an overview of the progress in scene image classification and presents related work on traffic scene image classification. Additionally, it introduces the fundamental principles of the Res2Net model, which serves as the baseline network in this paper. The Materials and Methods section outlines the methodology employed in this study. The Experiments section covers the datasets and experimental details. The Discussion section discusses the experiment results, including comparisons with existing models and an ablation study. The Failure Cases section discusses and analyzes the failure cases. Finally, the Conclusions section presents the conclusion.

## Related works

In the earlier stages of scene image classification, the predominant approach involved the utilization of manually designed feature extractors to capture diverse image-specific attributes, including color, shape, texture, and spectral information. Notable handcrafted descriptors include color histograms, texture features, Histogram of Oriented Gradients (HOG), and Scale-Invariant Feature Transform (SIFT). However, these methods relied heavily on domain expertise and experience, often necessitating the redesign and fine-tuning of feature extractors to suit different datasets and tasks. Various coding methods, including improved Fisher Kernel (IFK) [11], locally aggregated vector descriptors [12], Spatial Pyramid Matching (SPM) [13], and the famous Bag of Visual Words (BoVW) [14], were applied to capture the overall scene representation using these local descriptors. Although these methods have achieved some results, their classification performance could be improved, especially in complex scenes. The difficulty lies in adapting hand-designed features to different scenarios and tasks. Traffic scene images mainly encompass intricate features and high-level semantic information, making effective classification based on manually defined features challenging.

In recent years, machine learning based on probabilistic statistics has provided numerous viable approaches for image classification. Typical machine learning methods include Support Vector Machines, Decision Trees, k-means Clustering, Principal Component Analysis [15], and Sparse Representation [16]. However, these machine learning-based classification methods belong to shallow learning networks, thereby facing challenges in constructing complex function representations and exhibiting limited adaptability in classifying traffic scene images with intricate samples. This concern was raised by Anna Bosch et al. [17], who highlighted a notable issue with methods utilizing image features for scene classification—they often struggle to generalize beyond the training set to additional image data. Additionally, a critical drawback is the absence of intermediate semantic image descriptions, which hold substantial value in accurately determining scene types.

In 2006, Hinton et al. [18] proposed a Deep Belief Networks (DBN) model in deep learning and applied it to image classification tasks. Over the past few years, researchers have made significant progress in image classification tasks using deep learning methods. Initially, deep learning methods relied primarily on Convolutional Neural Networks (CNNs), which extract features through multiple convolutional and pooling layers. Inspired by the Deformable Part Model (DPM) [19], Liu et al. [20] proposed the use of CNN features for scene recognition, achieving excellent results on the Scene15 dataset [21]. Furthermore, their CNN-based method utilized a pre-trained model from ImageNet and fine-tuned it. As multiple objects and their states determine scenes, a network trained on ImageNet becomes more capable of recognizing objects in scenes, thereby improving the accuracy of scene recognition and classification.

With the development of various CNN models, notable examples being AlexNet [22], VGG [23], GoogleNet [24], and ResNet [25], significant performance improvements were achieved in image classification tasks at that time. Researchers gradually realized the impact of the depth and width of convolutional layers on network performance. Consequently, new feature extraction networks were proposed, such as Inception [26], MobileNet [27], and EfficientNet [28]. These networks employ different design strategies, such as parallel convolution, depth-wise separable convolution, and automated searching, to enhance network performance and efficiency.

Inspired by the conclusion that multi-level CNN feature fusion improves image classification capabilities from [29], Tang et al. [30] proposed a deep CNN model based on GoogLeNet called G-MS2F (multi-stage feature fusion), which validated the effectiveness of multi-level feature fusion. Gradually, scene image classification has also been applied to scene recognition in remote sensing images [31–34], achieving higher classification accuracy. Numerous studies have found that multi-scale features and attention mechanisms can improve the performance of deep learning networks. The DSMSA-Net proposed by Khan et al. [35] adopts an encoder-decoder structure and integrates attention mechanisms to handle road segmentation tasks in high-resolution satellite images. The encoder part of the network extracts multi-scale features from different convolutional layers. The decoder section consists of two modules: Scale Attention Unit (SaAU) and Spatial Attention Unit (SpAU). Among them, SaAU utilizes feature maps of different residual blocks in the encoder to extract multi-scale information, remove noisy areas, and focus on areas related to road semantic description. SpAU captures meaningful contextual information by integrating feature maps generated by average pooling and maximum pooling operations. This study also demonstrates the effectiveness of multi-scale features and spatial attention techniques in different scenarios. A relevant review paper [36] suggests that when the number of training samples is insufficient to train a new CNN from scratch, fine-tuning a pre-trained CNN on the target dataset yields better results than full training. This observation holds for all image classification tasks.

With the advancement of scene image classification, researchers have applied many optimized feature extraction networks to traffic scenes. Wu et al. [37] proposed a method that combines deep CNN and VLAD coding for the automatic recognition of traffic scenes. By leveraging the feature representation capability of CNN and the advantages of VLAD coding, the method effectively captures the features of traffic scenes and achieves satisfactory classification performance. Sikiri et al. [38] introduced a strategy for classifying traffic scenes under limited image representation resources, such as bandwidth and storage space. Their method primarily relies on image descriptor-based image classification, which demonstrates good classification performance. The paper also explores the challenge of traffic scene classification on datasets with unknown categories. It shows that even in the absence of target dataset labels, reliable classification results can be obtained using concise image descriptors. This is particularly important for real-world applications where adaptability to changing sets of categories is crucial. However, using image descriptors may result in the loss of detailed information, which may affect accuracy in certain scenarios. Shaik et al. [39] discuss the application of neural networks in predicting the severity of road accidents. While this differs from traffic scene image classification, it provides insights into the predictive power and accuracy of neural networks in the transportation field. The study demonstrates the potential of neural networks in processing traffic data and predicting traffic attributes. Ni, Jianjun et al. [40] fused local and global features for scene classification. Specifically, they employed an improved Faster RCNN network to extract features of representative objects in the scene, thereby obtaining local features. They introduced a novel residual attention module to emphasize local semantics related to the driving scene. Additionally, global features were extracted using an enhanced Inception module, which incorporated a hybrid Leaky ReLU and ELU function to reduce convolutional kernel redundancy and enhance robustness. The proposed method achieved superior performance in the task of self-driving car scene classification. However, the fusion of local and global features in this dual network form will increase complexity and computational costs. Z. Dorrani [41] presented an approach to traffic scene analysis and classification using deep learning and convolutional neural networks. The study utilized the ResNeXt architecture, a powerful framework that has garnered significant attention across various fields. The objective of the study was to classify traffic scene images into three categories: cars, bicycles (including both bicycles and motorcycles), and pedestrians. The application of deep learning in traffic scene classification was introduced, and the advantages of its high accuracy and efficiency were explored. The study provides a reference and benchmark model for traffic scene classification using deep learning methods. Bulbula Kumeda et al. [42] utilized a CNN network consisting of four convolutional layers and three fully connected layers for image classification on the Traffic-Net dataset. They achieved an impressive accuracy of 94.4% across four image categories: dense traffic, sparse traffic, fire, and traffic accidents. This study demonstrates the effectiveness of using CNN for image classification. A. K. Agrawal et al. [43] proposed a model based on ResNet50 and SVM, leveraging clustering and classification concepts for the binary task of detecting traffic accidents from surveillance camera frames. Their method achieved an accuracy of 94.14%. This study demonstrates the effectiveness of using CNN for image classification. Robles-Serrano et al. [44] extracted keyframes from traffic videos and employed a modified Inception V4 architecture to extract visual feature vectors for real-time classification and detection of traffic accidents. Furthermore [45], demonstrated the effectiveness of transfer learning using VGG16, GoogLeNet, and ResNet50 for classification tasks on the Traffic-Net dataset, achieving an accuracy of over 95%.

**Fig 1:**
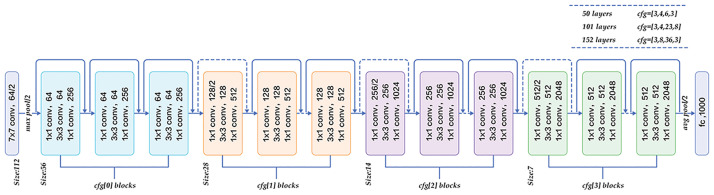
Illustration of the ResNet model architecture using ResNet-50 as an example.

In summary, from manually designed feature extractors, to coding methods, to probabilistic and statistical machine learning methods, to deep learning methods, existing methods and technologies for traffic scene image classification have continuously improved the accuracy and effectiveness of traffic scene image classification. Deep learning methods eliminate the limitations of manually designed features in traditional methods by automatically learning features and classifiers. Many important techniques for improving model performance, such as multi-scale features and attention mechanisms, have also been developed for deep learning networks. The application of deep learning methods to image classification tasks for traffic scenes is becoming more and more popular. However, compared to the rapid development of deep learning and its feature extraction networks, there are still many deep learning methods and strategies that have not been well applied to the field of traffic scene image classification. There are still challenges for complex scenes that require further research and improvement. This paper builds a model based on the Res2Net [46] feature extraction network. Res2Net is an improved network model based on Resnet. ResNet offers various depth options such as ResNet-18, ResNet-34, ResNet-50, ResNet-101, and ResNet-152. Among these options, ResNet-50 balances network depth and the ability to capture deep-level feature information. The prominent advantage of the ResNet series lies in addressing the problem of vanishing gradients during the training of deep neural networks. The ResNet series employs residual units, where the input and output of each unit consist of multiple convolutional layers and activation functions. However, a residual unit includes a skip connection that directly adds the input signal to the output signal, yielding the residual. The residual is then added to the output to obtain the final output. This residual calculation ensures unobstructed information flow from the forward to the backward propagation, thereby mitigating the problem of vanishing gradients and enabling deeper networks. Fig 1 illustrates the network architecture diagram for ResNet-50.

Res2Net enhances network performance by introducing a novel approach to handle multi-scale feature maps based on the ResNet architecture. Each ResNet block comprises several convolutional layers with identical receptive field sizes in ResNet. However, the feature maps at different layers may possess varying scales, leading to the potential loss of valuable information by individual convolutional layers. To overcome this limitation, Res2Net introduces the concept of “Split-Attention,” whereby the input feature maps are partitioned into multiple branches, each branch having a distinct receptive field size. This operation enables more effective capture of features across different scales. Res2Net decomposes the 3x3 convolution operation into multiple scales, followed by channel grouping through 1x1 convolutions.

**Fig 2:**
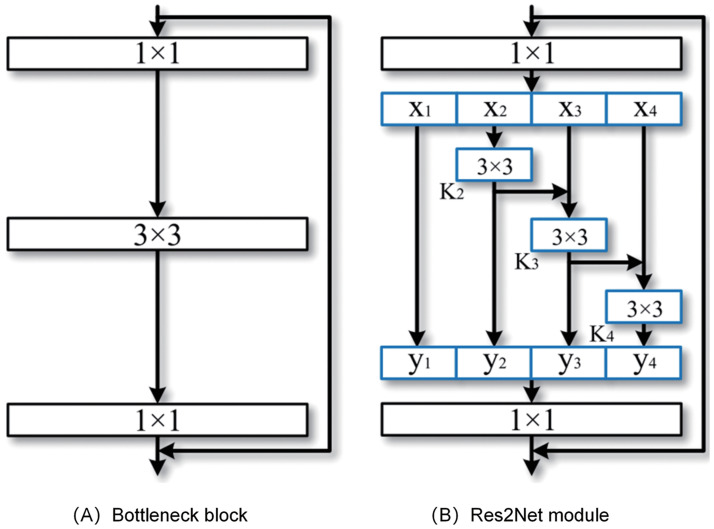
Comparison between the bottleneck block and the proposed Res2Net module. (A) Basic composition of the CNN network architecture. (B) Res2Net multi-scale module(the scale dimension s = 4).

Furthermore, a scale parameter denoted as “s” is introduced to control the number of channel groups. Consequently, each convolutional layer encompasses multiple receptive fields, enabling the acquisition of multi-scale features. Fig 2 illustrates the architecture of this Res2Net module.

In the Res2Net series, several prominent network architectures have been developed, including Res2Net-50-26w-4s, Res2Net-101-26w-4s, Res2NeXt-50, Res2Net-DLA-60, Res2NeXt-DLA-60, and Res2Net-v1b. The parameters “w” and “s” represent the width of the network and the number of scale divisions for the feature maps, respectively. Common configurations include 26w-4s, 48w-2s, 14w-8s, 26w-6s, and 26w-8s. Res2NeXt-50 is a network structure derived from Res2Net by incorporating the concept of grouped convolutions. Res2Net-DLA-60 combines Res2Net with DLA [47], utilizing 60 sub-feature maps for convolution and dividing the input feature maps into multiple scales. Res2NeXt-DLA-60 further applies grouped convolutions to the Res2Net-DLA-60 architecture for convolutional operations. Res2Net-v1b-50 and Res2Net-v1b-101 are improved versions based on Res2Net, employing the “split-transform-merge” strategy inspired by ResNeXt [48] and trained using the data augmentation technique CutMix. The Res2Net paper has demonstrated the superiority of their backbone networks over ResNet, ResNeXt, DLA, and other networks. It has achieved remarkable performance in image classification, object detection, semantic segmentation, and instance segmentation tasks. However, classifying traffic scene images poses challenges due to their complex semantic information and high category similarity. The feature extraction capability of Res2Net alone is limited, necessitating the integration of other models or methods to enhance the network’s feature extraction ability for improved accuracy and generalization in classification tasks.

## Materials and methods

### Adaptive Feature Refinement Pyramid Module(AFRPM)

**Fig 3:**
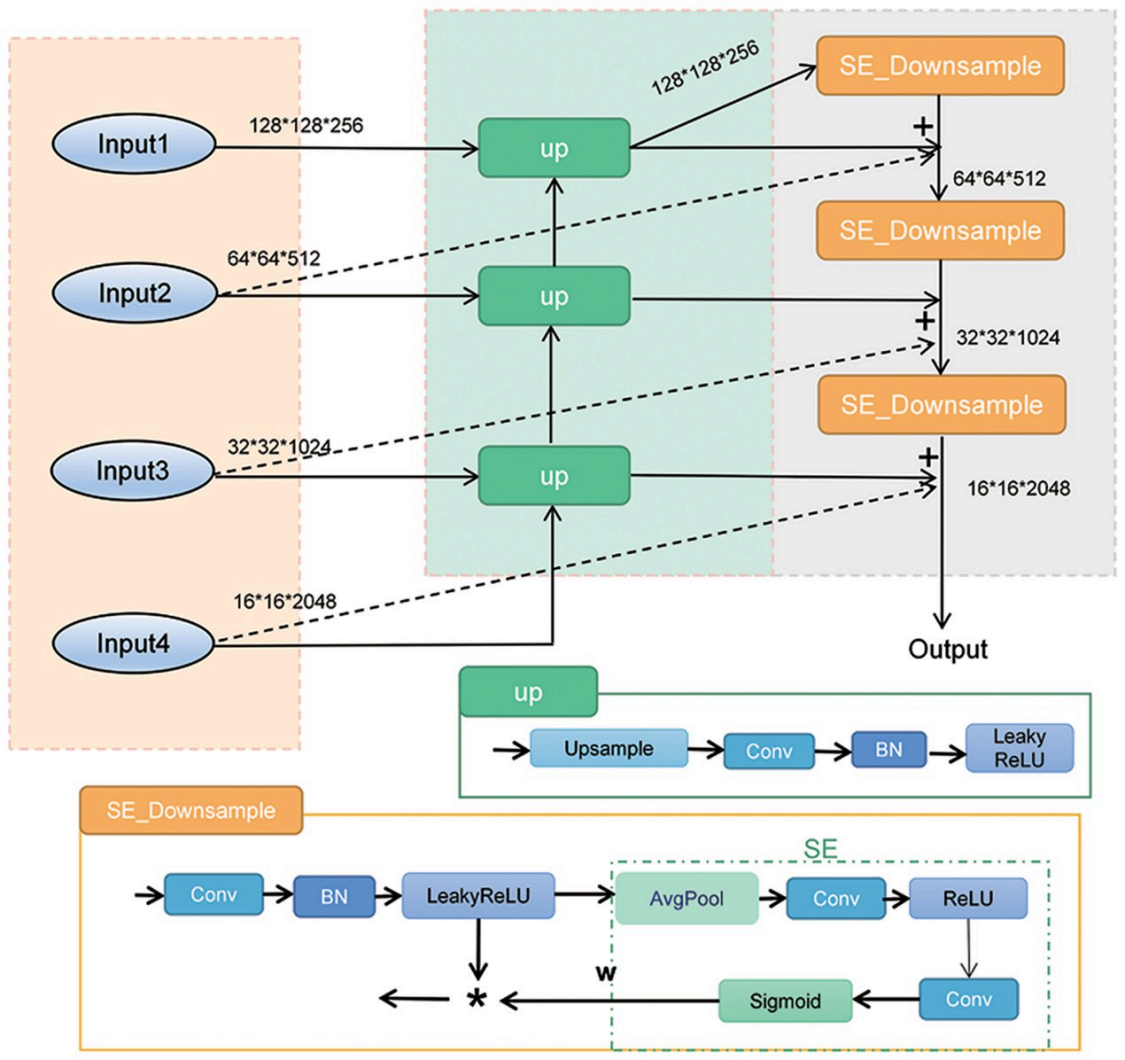
Structure of the Adaptive Feature Refinement Pyramid Module (AFRPM).

Building upon the FPN (Feature Pyramid Network) structure, we introduce the Adaptive Feature Refinement Pyramid Module (AFRPM) in Fig 3. In improving the multi-scale feature extraction capability of feature extraction networks, the effect of FPN structure is significant, and FPN has played a crucial role in detecting multi-scale targets in [49]. Although the FPN structure effectively incorporates high-level features to enhance the semantic content of lower-level features, it still exhibits certain limitations. For example, feature fusion in FPN can lead to information loss and resolution distortion. We draw inspiration from PANet’s [50] bottom-up path augmentation approach to overcome these challenges. We enrich the entire feature hierarchy by leveraging precise localization cues from lower-level features, reducing the information path between lower- and higher-level features. Moreover, we employ PANet’s adaptive feature pooling technique to construct the Adaptive Feature Refinement Pyramid Module, enabling adaptive feature optimization.

Before explaining the AFRPM module, let us introduce the UP and SE_DownSample sub-module. The operation within the UP submodule consists of two steps. First, the input feature map x undergoes an upsampling operation to obtain an upsampled feature map. Second, the upsampled feature map undergoes a series of operations, including convolution, batch normalization, and LeakyReLU activation, resulting in the output feature map. The UP sub-module enables the upsampling of low-resolution feature maps to match the resolution of high-resolution feature maps, thereby preserving more spatial information. Additionally, the UP sub-module enhances the model’s expressive power and performance by performing a series of operations, such as convolution, batch normalization, and activation functions, on the upsampled and input feature maps. This upsampling and feature fusion structure helps the model capture fine details and texture information in images, thereby improving the model’s visual perception and accuracy.

In the SE_DownSample sub-module, the operations of the Squeeze-and-Excitation module [8] consist of three steps. First, the input feature map x undergoes a 3x3 convolution operation, simultaneously increasing the number of channels to twice its original size. It is then normalized and passed through the LeakyReLU activation function to obtain the feature map y. Second, through adaptive average pooling, followed by a series of operations including convolution, batch normalization, activation functions, and sigmoid, a weight vector w is obtained. The weight vector calculates the importance of each channel in the feature map y. Finally, the input feature map y is multiplied element-wise with the weight vector w to obtain the output feature map z. Specifically, the following equation represents the operations of the SE module:

$$\begin{array}{c} y=f_{\text{LRelu}\;}f_{\text{BN}}(f_{s=2}^{3\times 3}\left(x\right)) \end{array}$$ (1)

$$\begin{array}{c} w=\sigma (W_{2}(\text{Relu}(W_{1}(\frac{1}{H*w}\sum_{i=1}^{W}\sum_{j=1}^{H}u_{c}\left(i,j\right))))) \end{array}$$ (2)

$$\begin{array}{c} z=w\times y \end{array}$$ (3)

In the formula, $f_{\text{s}=2}^{3\times 3}$ represents a 3x3 convolution operation with a stride of 2, *σ* represents the Sigmoid function, × represents element-wise multiplication, 1/*H* * *W* represents the adaptive average pooling operation applied to the input feature map u. *W*_1_ and *W*_2_ represent convolutional layers.

The SE module enables adaptive adjustment of the importance of each channel in the input feature map, thereby enhancing the model’s focus on essential features while reducing interference from irrelevant features. This adaptive characteristic helps the model better capture multi-scale features and information from the input image. In the main structure of Res2Net, there are four layers. The outputs of the second, third, and fourth layers are sent to the UP sub-module for upsampling and halving the channel size, followed by element-wise addition with the previous layer’s output. These operations correspond to the middle section of the feature pyramid depicted in Fig 3. The resulting outputs are then fed into the SE_DownSample sub-module for downsampling and channel-wise attention-weighted processing, following the order of three consecutive SE_DownSample operations shown in the right section of Fig 3. The feature maps obtained from the SE_DownSample module and the UP sub-module, along with the feature maps extracted from the second, third, and fourth layers, are summed together based on the criterion of having identical size and channel numbers. This fusion of feature maps from these three operations produces a more comprehensive representation of the feature map.

### Frequency Domain Attention Module(FDAM)

In the context of traffic scenes, RGB spatial domain images may not fully exploit the information contained within the image. Certain essential features like vehicle states and pedestrians often exhibit specific frequency distribution patterns. Frequency domain attention can effectively capture these patterns and enhance the model’s focus on these features, thereby improving the model’s performance. Furthermore, frequency domain attention can help suppress high-frequency noise and low-frequency background interference, thereby enhancing the model’s robustness. Specifically, frequency domain attention leverages the Fast Fourier Transform (FFT) to obtain the energy spectrum of the input image. It utilizes this spectrum for channel-wise fusion, enhancing the model’s attention to input features. The FFT is an efficient and fast computational method for calculating the Discrete Fourier Transform (DFT) using computers. DFT is a transformation that converts a discrete-time domain signal into its frequency domain representation. The discrete Fourier transform is a linear transformation, and its inverse transform is reversible when the sequence length is prime or when the sequence length and the factors of the sequence length are coprime. The formulas for the discrete Fourier transform and the inverse discrete Fourier transform are as follows:

$$\begin{array}{c} x_{n}=\sum_{k=0}^{N-1}X_{k}e^{-i\frac{2\pi }{N}\text{kn}},n=0,\ldots ,N-1 \end{array}$$ (4)

$$\begin{array}{c} X_{k}=\frac{1}{\;N}\sum_{k=0}^{N-1}x_{n}e^{i\frac{2\pi }{N}\text{kn}},k=0,\ldots ,N-1 \end{array}$$ (5)

In Eqs (4) and (5), *x*_*n*_ represents the frequency-domain representation of a discrete signal, N denotes the length of the sequence, *X*_*k*_ represents the discrete signal sequence in the time domain, i represents the imaginary unit, and k and n are the indices in the frequency domain and time domain, respectively. The essence of these equations is that for a discrete time-domain signal *X*_*k*_ of length N, it can be decomposed into a linear combination of N complex exponentials. Each complex exponential, characterized by the exponent term ${\text{e}}^{-\text{i}\frac{2\pi }{\text{N}}\text{kn}}$, represents the amplitude and phase of the signal at the frequency n/N. Therefore, *x*_*n*_ represents the amplitude and phase of the signal at n/N in the frequency domain.

**Fig 4:**
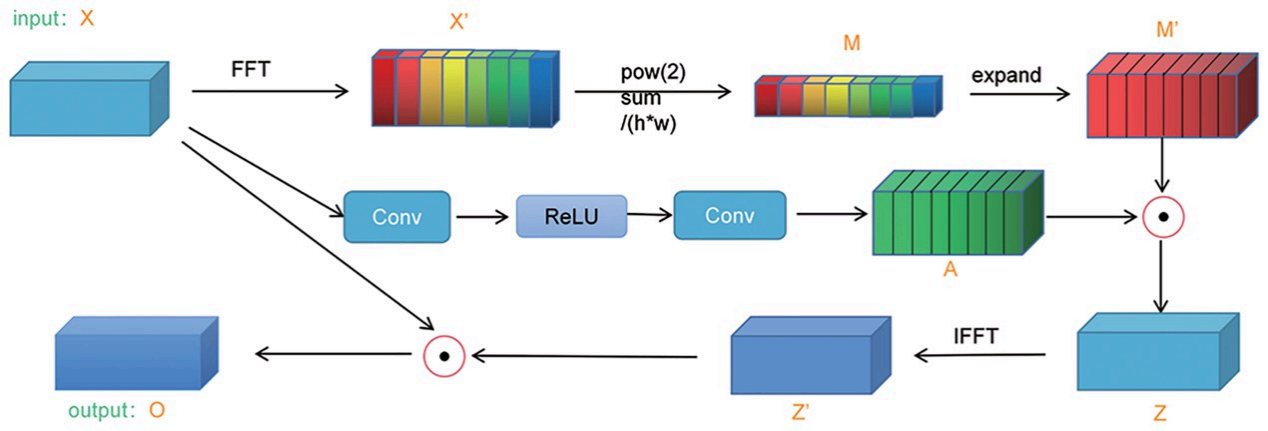
Structure of the Frequency Domain Attention Module (FDAM).

The frequency-domain attention module employed in our model leverages the Fast Fourier Transform (FFT) to acquire the frequency energy spectrum of the input feature x. This technique effectively amplifies significant feature representations and facilitates the weighted fusion of energy spectra through channel attention coefficients, enhancing the model’s focus on input features. Fig 4 graphically depicts the operational workflow of this attention mechanism.

An input tensor X with dimensions H * W * C undergoes a fast Fourier transform, generating a tensor X’ of size H * W * C, wherein each channel represents an energy spectrum associated with a specific frequency. The energy spectrum calculates the squared summation of magnitudes in the frequency domain for each channel, subsequently normalizing it by dividing the total number of pixels in the feature map. Spatial averaging is applied to the energy spectra along the spatial dimensions, yielding a tensor M with dimensions 1 * 1 * C. The value M_c in the c-th channel of M denotes the average energy spectrum for that particular channel. To perform a weighted fusion of M with the input tensor X, M is expanded into a tensor M’ with the exact dimensions as X. This expansion entails duplicating M across the spatial dimensions, resulting in a tensor M’ with dimensions H * W * C. Next, feature extraction is performed on the input tensor X along the channel dimension, yielding a tensor with dimensions H * W * C/r, where r represents a reduction ratio conventionally set to 16. Subsequently, two 1 * 1 convolutional kernels are employed to process the tensor, generating a tensor A with dimensions H * W * C. Each channel within A corresponds to a channel attention coefficient denoting the relative importance of the respective channel. Element-wise multiplication is then performed between A and M’, resulting in a tensor Z with dimensions identical to the input tensor X. Finally, Z undergoes an inverse fast Fourier transform, extracting the fundamental part to yield a tensor Z’ with the same shape as X. Each channel within Z’ corresponds to a weighted energy spectrum derived from the fusion of channel attention and spatially pooled energy spectra. The element-wise multiplication between the input tensor X and the tensor Z’ defines the ultimate output tensor O.

### Spatial-Channel and Similarity Attention Module(SCSAM)

Channel and spatial attention mechanisms are widely employed in deep learning, playing pivotal roles in improving model performance and feature extraction. This conclusion was also confirmed in [49]. In this study, we adopt the convolutional channel attention module and local spatial attention module proposed in [51], as well as the SimAM (Similarity Attention Module) introduced in [9], to construct the Spatial Channel and Similarity Attention Module (SCSAM). The SCSAM module encompasses a dual channel attention module (DCAM), a multi-scale pooled spatial attention module (MSPAM), and the SimAM module. By integrating these components, our algorithm offers comprehensive enhancements across channel, spatial, and neuron dimensions.

#### Dual Channel Attention Module(DCAM)

**Fig 5:**
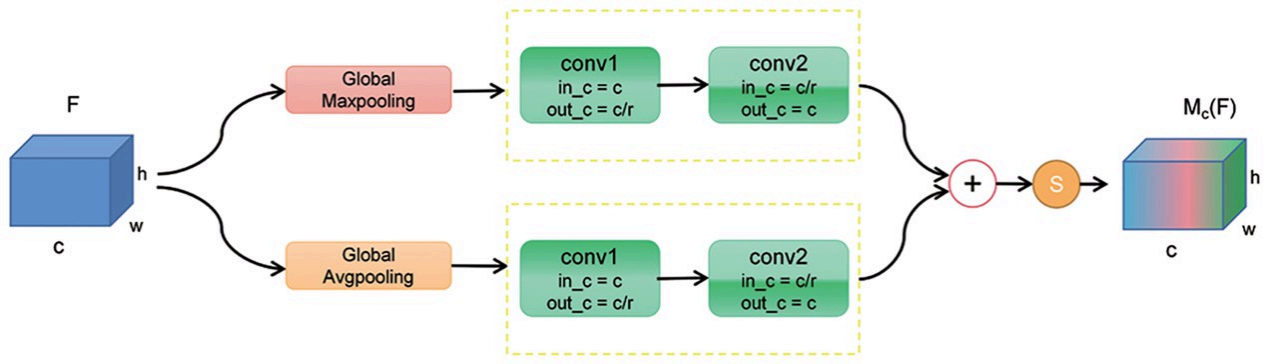
Structure of the Dual Channel Attention Module (DCAM).

By learning channel attention weights, the model can automatically adjust the importance of different channels, emphasizing task-relevant features while reducing attention to irrelevant or noisy features. This mechanism improves model expressiveness and generalization capabilities, enhancing its overall performance. Additionally, the channel attention module’s adaptive selection of attended channels reduces unnecessary computational and storage costs, enhancing model efficiency and speed. This study proposes an alternative structure for the channel attention module, the Dual Channel Attention Module (DCAM), as illustrated in Fig 5.

Initially, assuming an input feature map F with dimensions H × W × C, global average pooling and global maximum pooling operations are applied to reduce the feature map’s spatial dimensions to 1 × 1, resulting in a 1 × 1 × C feature map. Subsequently, these two pooled feature maps are individually processed by 1 × 1 convolutional layers to reduce the channel dimension to 1/r of the original size. Another 1 × 1 convolutional layer can restore the channel dimension to its original size, resembling a Multilayer Perceptron (MLP) mechanism. Here, the parameter r represents the compression ratio conventionally set to 16. The feature maps obtained from the two convolutional operations add to yield a novel feature map. Finally, the feature map is activated using the sigmoid activation function, resulting in the output feature map Mc(F). Hence, the formulation for this module takes the following expression:

$$\begin{array}{c} M_{c}\left(F\right)=\sigma (f_{c/r\to c}^{1\times 1}(f_{c\to c/r}^{1\times 1}(\;F_{\text{max}}^{c})+f_{c/r\to c}^{1\times 1}(f_{c\to c/r}^{1\times 1}(\;F_{\text{avg}}^{c})) \end{array}$$ (6)

In the formula, $f_{\text{c}/\text{r}\to \text{c}}^{1\times 1}$ and $f_{\text{c}\to \text{c}/\text{r}}^{1\times 1}$ represent two 1 × 1 convolutional operations, ${\text{F}}_{\text{max}}^{\text{c}}$ and ${\text{F}}_{\text{avg}}^{\text{c}}$ respectively denote the feature maps obtained after global maximum pooling and global average pooling, and *σ* represents the sigmoid activation function.

The original work [51] concatenates the two feature maps along the H dimension and employs a 2x1 convolutional layer to merge the global feature maps while performing channel-wise weighting. However, this approach introduced only a single transformation and a set of parameters. By utilizing two separate 1×1 convolutional layers, the module generates a more significant number of intermediate feature maps, enabling each convolutional layer to learn distinct feature representations. This enhanced design promotes module flexibility, allowing for learning diverse attention weights across different dimensions. Furthermore, adopting two 1x1 convolutional layers endows each layer with independent weights and non-linear transformations, facilitating a more comprehensive capture of inter-feature relationships and discrepancies.

#### Multi-Scale Pooling Spatial Attention Module(MSPAM)

**Fig 6:**
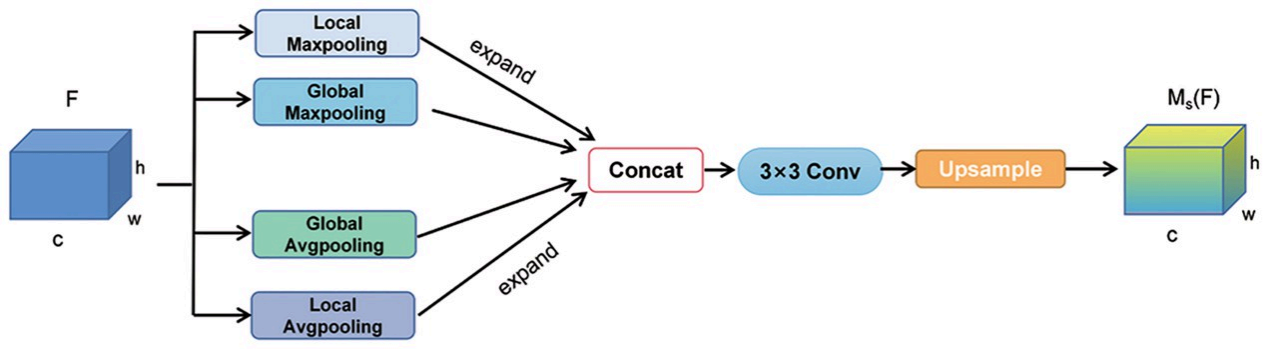
Structure of the Multi-Scale Pooling Spatial Attention Module (MSPAM).

Not all regions within an image contribute uniformly to the classification task. It is imperative to concentrate solely on the pertinent areas that bear relevance to the classification task, such as the vehicle traffic flow and road information in the context of traffic scene image classification, while disregarding the less significant information adjacent to the road. The spatial attention module plays a pivotal role in identifying and processing the most pivotal regions within the network, which bears significant importance when dealing with traffic scene image data exhibiting dynamic backgrounds, occlusions, or noise. Hence, in this paper, we introduce a Multi-Scale Pooling Spatial Attention Module (MSPAM) within the framework of SCSAM, as illustrated in Fig 6.

Assuming an input feature map F with dimensions of H×W×C, we employ localized and global maximum pooling and localized and global average pooling. By employing the expand function, we can subsequently expand the resulting feature maps obtained from localized maximum pooling and localized average pooling operations to match the size of the original feature map (H×W). These four feature maps are then concatenated along the channel dimension and fed into a 3×3 convolutional layer for feature extraction. Lastly, the module applies nearest-neighbor interpolation for upsampling, resulting in the ultimate feature map denoted as Ms(F). Thus, the formulation for this module takes the following expression:

$$\begin{array}{c} M_{s}\left(F\right)=f_{n}(f^{3\times 3}[\;F_{\text{max}}^{G};F_{\text{avg}}^{G};f_{\text{ex}}(F_{\text{max}}^{L});f_{\text{ex}}(F_{\text{avg}}^{L})]) \end{array}$$ (7)

In the formula, ${\text{F}}_{\text{max}}^{\text{G}}$ and ${\text{F}}_{\text{avg}}^{\text{G}}$ correspond to the feature maps resulting from global maximum pooling and global average pooling, respectively. ${\text{F}}_{\text{max}}^{\text{L}}$ and ${\text{F}}_{\text{avg}}^{\text{L}}$ signify the feature maps obtained from local maximum pooling and local average pooling, respectively. *f*^3×3^ represents the application of a 3 × 3 convolutional operation, while *f*_n_ denotes the operation of nearest-neighbor interpolation employed for upsampling.

In contrast to the local spatial attention module described in [51], the module under consideration does not incorporate a mechanism to dynamically adjust the importance of different positions in the feature map through learned spatial attention weights. However, it employs a multi-scale feature fusion approach. The MSPAM module captures local and global information at multiple scales by leveraging pooling operations and feature concatenation. It integrates them, facilitating a comprehensive understanding and representation of the input image features. Experimental validation confirms the suitability of this multi-scale pooling spatial attention module for the utilized traffic scene image dataset.

#### SCSAM: Combination of DCAM, MSPAM, and SimAM

**Fig 7:**
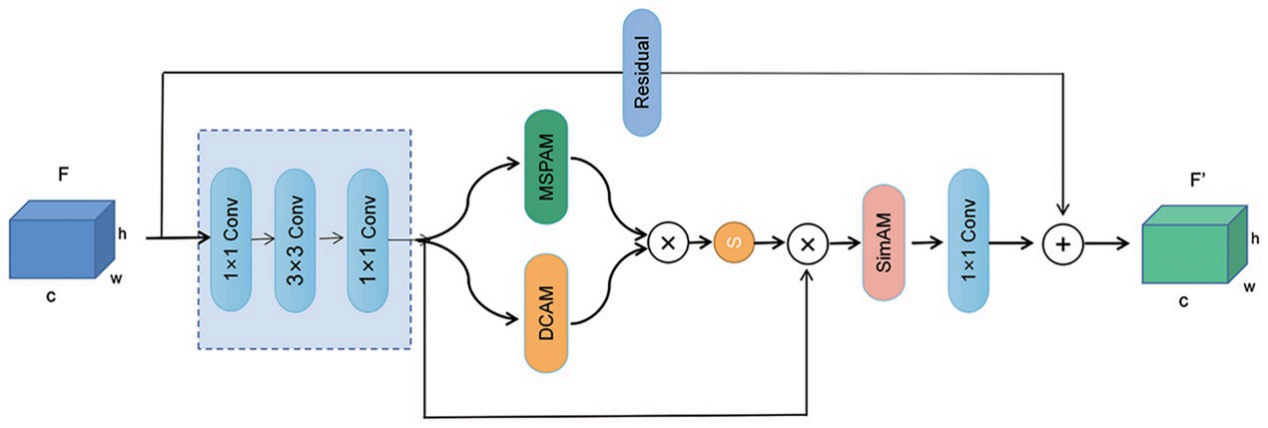
Structure of the Spatial-Channel and Similarity Attention Module(SCSAM).

SimAM, proposed in [51], is a similarity-based attention module that can directly infer three-dimensional attention weights within the network layers without introducing additional parameters. This module draws inspiration from well-established neural science theories, utilizing optimization of an energy function to determine the importance of each neuron. The outputs of DCAM and MSPAM are multiplied and serve as the input to SimAM. Specifically, DCAM and MSPAM compute complementary channel and spatial attention in parallel. After applying sigmoid activation, SimAM performs similarity-based weighting on these attention maps to obtain the final feature map. Fig 7 illustrates the specific architecture of SimAM. The concatenated and parallel structure effectively leverages different attention mechanisms, enhancing the expressive power of essential features and improving the model’s perception of features at different scales and correlations. Experimental results demonstrate that this architecture achieves superior classification performance in traffic scene image classification.

### Network structure

**Fig 8:**
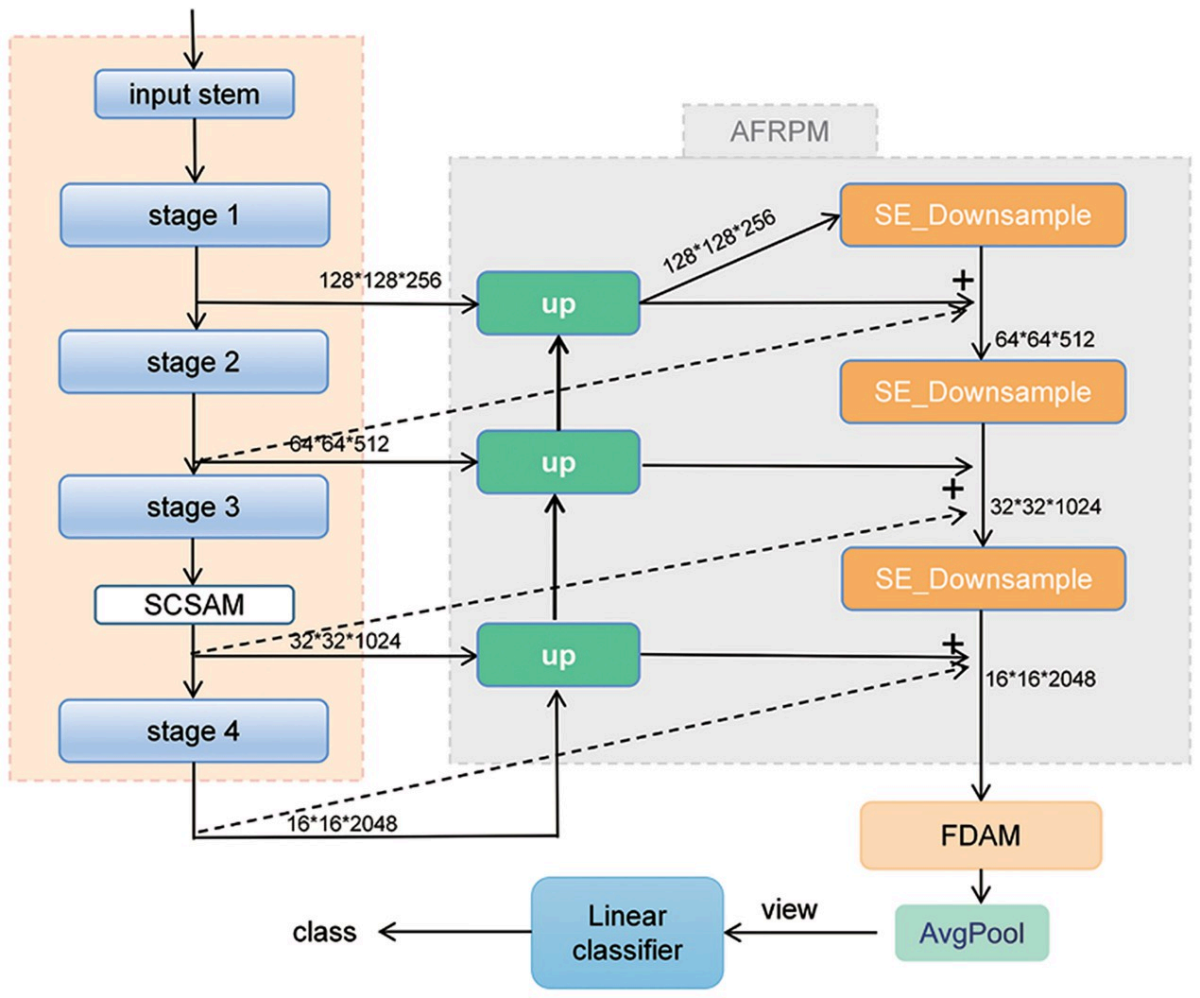
The overall architecture of the proposed multi-scale and multi-attention model for classifying traffic scene images based on Res2Net_v1b.

As mentioned in the Related Works section, this study adopts the Res2Net_v1b model from the Res2Net series due to its well-designed architecture, excellent performance, and low model complexity. Moreover, Res2Net_v1b has readily available pre-trained models on ImageNet, facilitating the implementation of the proposed model. Fig 8 illustrates the architecture of the proposed model in this study. Experimental results reveal that placing the SCSAM (Spatial Channel-Separated Attention Module) on the third-layer outputs of Res2Net yields superior performance. Res2Net is a multi-scale feature extraction network that enhances the expressive power of the network by introducing multiple branches within each residual block. Compared to the features from the first and second layers, the third-layer features have undergone multiple branch fusions, resulting in richer semantic information and more vital feature representation capabilities. Considering issues such as resolution downsampling, sparse semantic information, and computational costs, the low-resolution feature maps from the fourth level are not conducive to applying spatial attention. The third-layer outputs typically possess higher resolution, preserving more detailed information, which enables spatial and channel attention mechanisms to better capture and adjust critical information within the feature maps. The fourth-layer features usually have higher resolution and more channels, increasing computational complexity and extended training and inference times. Therefore, the SCSAM module achieves optimal performance when taking the third-level outputs of Res2Net as inputs. The Adaptive Feature Refinement Pyramid Module (AFRPM) inputs the outputs from each level of Res2Net to further extract multi-scale features. These features undergo processing using Fourier domain attention to capture frequency domain distribution patterns, resulting in the acquisition of the final output. Subsequently, the classifier receives the final output to complete the classification task.

## Experiments

### Dataset and experimental configuration

The dataset used in this experiment is the Traffic Net Dataset V1 dataset provided by OlafenwaMoses. The reason for the selection of this dataset is that it has a number of advantages for the classification task of traffic scene images. Firstly, it covers different traffic scenarios. These include roads, intersections, car parks, etc. The images in the dataset have different lighting conditions, weather conditions and perspectives. This allows the Traffic Net dataset to provide a more comprehensive and diverse range of traffic scenarios. This helps our model to learn and generalise better to different scenarios in practical applications. Secondly, due to the complexity and specificity of traffic scenarios, there are relatively few publicly available traffic datasets. Therefore, when selecting a dataset, we consider the specificity of traffic scene image classification. Currently, Traffic Net is a relatively comprehensive dataset that is publicly available. Finally, our selection of the Traffic Net dataset is also based on the support and precedents of other research. Some previous research has used the Traffic Net dataset in traffic scene image classification tasks and achieved certain results. These research results indicate that the Traffic Net dataset is feasible and effective in traffic scene image classification tasks. This dataset contains four types of traffic scene images, namely congested traffic, sparse traffic, traffic accidents and fires. The detailed composition of this dataset is shown in Table 1.

**Table 1:** Detailed composition of the dataset.

Dateset	Total	Type	Quantity
Traffic-Net	4400	Fire	1100
		Accident	1100
		Dense_Traffic	1100
		Sparse_Traffic	1100

## Image preprocessing and experimental details

**Fig 9:**
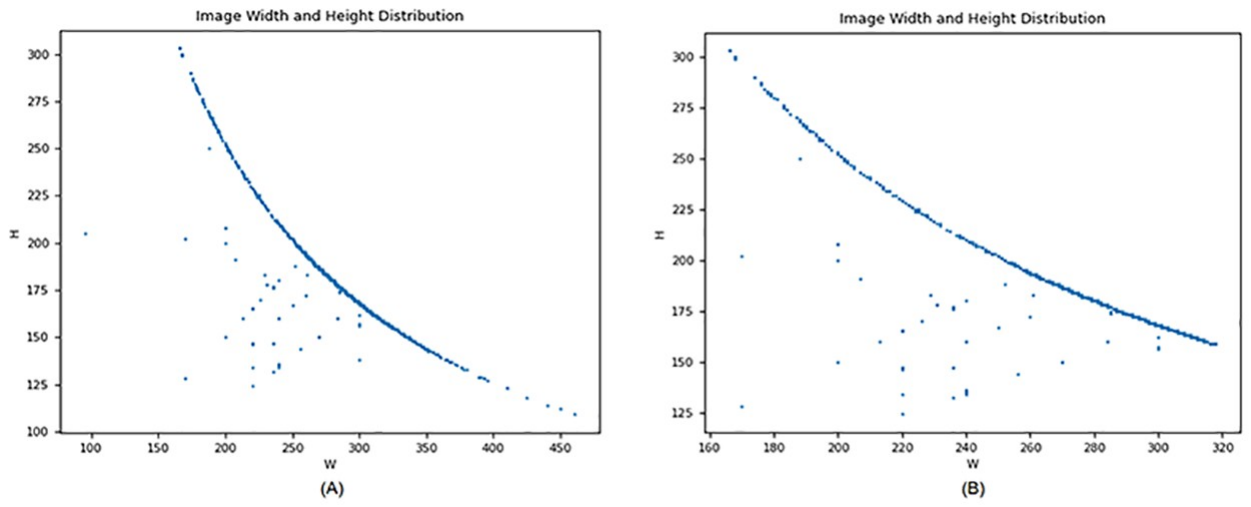
Scatter plot of dataset dimensions. (A) Scatter plot before filtering operation. (B) Scatter plot after filtering operation.

The image dataset was preprocessed by applying a filtering operation to remove images with inadequate dimensions or aspect ratios. This operation aimed to ensure that subsequent analysis or model training would not be negatively affected by images that did not meet the required size or proportion criteria. To accomplish this, a Python script utilizing the PIL library (Pillow) was employed. The filtering process involved three specific criteria: a minimum dimension of 100 pixels, a maximum dimension of 1000 pixels, and a minimum acceptable aspect ratio of 0.5. Images failing to meet any of these criteria were considered unsuitable and subsequently eliminated from the dataset. The scatter plot of data width and height before and after filtering operation is shown in the Fig 9.

In our study, we performed several preprocessing operations on the dataset during the image-loading phase. Firstly, to ensure consistent input sizes for deep learning models and avoid performance discrepancies, we resized all images to dimensions of 224x224 pixels, 448x448 pixels, and 512x512 pixels. Through experimentation, we found that the image size of 448×448 pixels yielded the optimal balance between overall speed and model performance. Secondly, in traffic scenes, objects and vehicles can have various orientations and directions. Specifically, in scenarios involving vehicle accidents, it is crucial to consider the surrounding environmental information rather than simply detecting vehicle flips. To introduce diversity into the dataset, we applied random horizontal and vertical flips during the data loading process. These flips simulate different orientations and enable the model to learn representations of objects from various directions. By incorporating random flips, we aimed to enhance the model’s generalization ability. Lastly, the Traffic-Net dataset comprises images captured under different lighting conditions, weather conditions, and colour features. To mitigate these variations, we standardized the images using the dataset’s mean and standard deviation. This normalization process helps eliminate redundant information and enables the model to focus more on important features. Standardization enhances the model’s perception capability and robustness. We randomized the order of the dataset using the random. shuffle function and allocated an 8:2 ratio for the training and test sets.

In our experiments, we observed that the Adam optimizer is more prone to overfitting our model and the complex traffic scene dataset. Its adaptive nature leads to minimal fluctuations in gradients during training, potentially resulting in overfitting to the training data. In contrast, the SGD optimizer with manual learning rate decay demonstrated better training performance on this dataset. Hence, we employed SGD as our network optimization algorithm, setting the momentum to 0.5. When using the SGD optimizer, we experimented with different learning rates and recorded the corresponding classification accuracies. The results of this experiment are presented in Table 2. Our results revealed that the choice of learning rate had varying effects on the classification accuracy. We achieved the highest accuracy of 96.88% in our specific image classification task and dataset by using a learning rate of 1e-4. This finding suggests that the model performed optimally with this learning rate. However, increasing the learning rate to 3e-4 led to a slight decline in accuracy to 96.23%. This decrease may be attributed to the higher learning rate causing the model to skip some local optima during training, thereby impacting performance. On the other hand, significantly reducing the learning rate to 1e-5 resulted in a notable decrease in classification accuracy to 91.88%. This reduction occurred because the excessively low learning rate led to slow convergence, hindering the model’s ability to adequately learn and adjust parameters. It is worth noting that setting the learning rate to 1e-3 resulted in gradient explosions, where the gradient values became exceptionally large during the backpropagation process, rendering the model unable to update parameters effectively. Therefore, selecting an appropriate learning rate is crucial for training and optimizing model performance in the SGD.

**Table 2:** Comparison of different learning rates.

Accuracy(%)	Learning rates
91.88	0.00001
96.23	0.0003
96.88	0.0001
-(Gradient Explosion)	0.001

In our study, we adopted the common cross-entropy loss function as the objective function for image classification tasks. The cross entropy loss function is a commonly used supervised learning loss function that measures the difference between predicted results and true labels. It is widely used in image classification tasks and has been shown to perform well in many deep learning models. Specifically, for each input image, our model first extracts the feature representation of the image using a convolutional neural network. We then feed these features into the fully connected layer, which maps the features to outputs representing the probabilities of each category through the softmax function. The actual label represents the actual category of the image. The cross-entropy loss function measures the prediction accuracy of the model by calculating the difference between the predicted probability and the true label. Mathematically, given the predicted probability distribution pi of a training sample and the probability distribution yi of the true label, the cross-entropy loss function can be defined as follows:

$$\begin{array}{c} L=-\sum yi*\text{log}(pi) \end{array}$$ (8)

In the formula, ∑ represents the sum of all categories and log represents the natural logarithmic operation. The smaller the result of the calculation of this loss function, the closer the predicted result of the model is to the true label, i.e. the higher the classification accuracy of the model.

To leverage prior knowledge and expedite the training process, we utilized pre-trained Res2Net weights as the initial parameters for our model. These weights were pre-trained on the extensive ImageNet dataset and possess excellent feature extraction capabilities. By incorporating these pre-trained weights, our model can benefit from the learned features, accelerate convergence, and achieve superior performance in classifying traffic scene images. We implemented the proposed framework using the Python library. The experimental setup involved the utilization of an NVIDIA TITAN RTX GPU with 24GB of graphics memory and an Intel (R) Xeon (R) CPU E5-2680 v3 @ 2.50GHz. We conducted 100 training epochs with a batch size of 16, ensuring sufficient iterations for model optimization and learning.

### Comparative experiment

We evaluated and compared several classical models on our dataset, and the experimental results are presented in Table 3. These models were trained and tested on our dataset, and various evaluation metrics, including accuracy, precision, recall, and F1 score, were computed. Accuracy is the most intuitive metric, representing the proportion of samples correctly predicted by the model. It is calculated by dividing the number of correctly predicted samples by the total number of samples. Precision measures the accuracy of the model in predicting positive class samples. It is calculated as the ratio of true positives (samples correctly predicted as positive) to the sum of true positives and false positives (samples incorrectly predicted as positive). A higher precision indicates fewer errors in predicting positive class samples. Recall measures the ability of the model to identify positive class samples. It is calculated as the ratio of true positives to the sum of true positives and false negatives (samples incorrectly predicted as negative). A higher recall indicates better identification of positive class samples. F1 Score is a comprehensive evaluation metric that balances precision and recall. It is the harmonic mean of precision and recall. The formula is as follows:

$$\begin{array}{c} F_{1}=2\cdot \frac{precision\cdot recall}{precison+recall} \end{array}$$ (9)

**Table 3:** Experimental results of different network models on the dataset.

Model	Accuracy(%)	Precision(%)	Recall(%)	F1(%)
Res2net50 [46]	94.89	94.89	94.89	94.88
Res2netxt50	95.03	95.14	94.96	94.98
Res2netv1b	95.05	95.04	95.05	95.04
ViT [52]	95.79	95.80	95.79	95.79
CNN [42]	94.43	94.47	94.54	94.43
VGG19_bn [53]	94.94	94.93	94.45	94.46
DenseNet121 [54]	92.15	92.09	92.16	92.21
GoogLeNet [24]	93.76	93.79	93.73	93.73
ResNet50 [25]	95.34	95.34	95.34	95.34
Resnet34	93.86	93.91	93.86	93.86
ResNet152	95.88	95.91	95.85	95.85
SE-ResNeXt [55]	95.06	95.05	95.03	95.02
MobileNetV3 [56]	91.14	91.02	91.01	91.02
shufflenetV2 [57]	91.09	91.11	91.08	91.09
Proposed	**96.88**	**96.84**	**96.88**	**96.80**

The F1 score ranges from 0 to 1, with higher values indicating a better balance between precision and recall. These evaluation metrics provide a comprehensive assessment of the model’s performance and support reliable quantitative analysis for our research.

The accuracy of our proposed model is high compared to the lightweight models MobileNetV3 and ShuffleNetV2. This is because the lightweight network sacrifices some of its performance to reduce the model size and speed up the inference, and cannot adapt to complex traffic scenarios. Meanwhile, we observe that in the ResNet series, the accuracy improves as the model depth increases. This is because the deeper the network depth, the larger the sensory field, and the feature information of large targets can be fully extracted. In this traffic scene dataset category, the features tend to be large targets, especially in the traffic accident scene and fire scene, where the accident vehicles and flames make up the majority of the image. However, the deeper the network depth is, the coarser the location information is at the same time, and for the two categories of dense and sparse traffic, the vehicle location information becomes important, which confirms that the deeper the network layers are, the better the detection effect does not get. Therefore, our model balances the characteristics of deep semantic and location information by using the network depth of res2net50, while adding a multiscale module and an attention module to synthesise high-level semantics with low-level texture and location information. VGG19_bn is a classical convolutional neural network and has a deep network structure, DenseNet121 is a densely connected convolutional neural network that facilitates the flow of information by connecting the feature maps of the previous layers with those of the layers behind. However, VGG19_bn uses a simple structure of stacked convolutional layers, and DenseNet121’s structure is more fixed, and both networks lack some effective attention mechanisms or location-aware modules, which may limit their performance when dealing with complex traffic scenarios. ViT mainly models feature relationships in images through global self-attention mechanisms, but in traffic scenes, local contextual information is very important for classification tasks. For example, in a dense traffic scene, the density and relative position between vehicles can provide important features. Different categories in a traffic scene may have different scale features. For example, a fire scene may contain small and localised flames, while a congested traffic scene may contain large and dense vehicles. Therefore, our Adaptive Feature Refinement Pyramid module is better at image classification for traffic scenes. This paper, also published on the Traffic-Net dataset, proposes a CNN structure consisting of four convolutional layers, three fully connected layers and a maximum pooling operation to progressively extract features and perform classification. It uses a 3x3 convolutional filter and a 2x2 pooling filter with ReLU activation function and batch normalisation. The depth and width of this CNN network limits the feature expressiveness of the network, and the model has no explicit mechanism for modelling global information. Due to the use of pooling operations, the network loses some of the detail information after each pooling layer and therefore cannot adequately capture the global contextual information of the entire image. From the quantitative evaluation metrics in Table 3, our model outperforms the other reference methods on this dataset in terms of accuracy, precision, recall and F1 score, and is about 1% higher than the most effective ViT model. Our proposed method outperforms all the reference methods by about 5% over the lightweight model and 2% over the baseline model.

**Fig 10:**
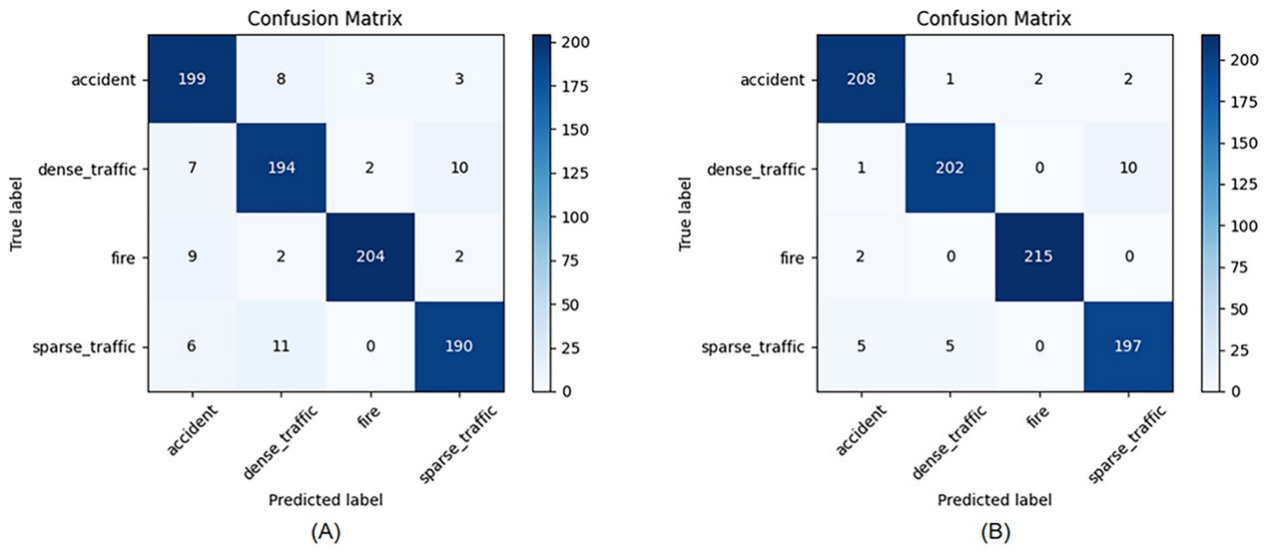
The confusion matrix between baseline model (A) and our proposed model (B) on the test set.

The confusion matrix in Fig 10 shows that our method is better at classifying categories with high feature similarity in distinguishing between them compared to the baseline.The two confusion matrices in the figure were generated with the weights of the baseline model and the weights of our proposed model on 850 test set images, respectively. It can be seen that our proposed model, which reduces the confusion between congested traffic and traffic accident detection, has a higher classification error for both sparse traffic and congested traffic, and the number of classification errors has also decreased.

### Ablation experiments

**Fig 11:**
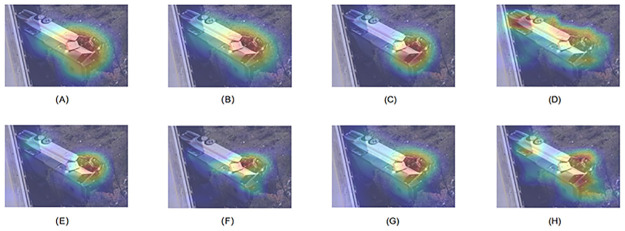
Comparative Visualization of CAM Heatmaps in the Last Layer for Different Models: (A) Res2Net Baseline, (B) Res2Net+AFRPM, (C) Res2Net+SCSAM, (D) Res2Net+FDAM, (E) Res2Net+SCSAM+FDAM, (F) Res2Net+AFRPM+FDAM, (G)Res2Net+AFRPM+SCSAM and (H) Proposed Approach.

In order to evaluate the effectiveness of the three models we proposed, we conducted module ablation experiments in different settings. For the ablation experiments, we still used the Traffic Net dataset, and the results are shown in Table 4. The Adaptive Feature Refinement Pyramid Module directly improves the classification accuracy and F1 score of the model by approximately 1%. This module combines Feature Pyramid Network (FPN) and Attention Mechanism (SE module). As early as in paper [7], it has been proven that the feature pyramid structure can fuse feature maps of different levels, better extract features of target objects of different sizes and proportions. In traffic scene images, because the same category can have multiple field of view angles and ranges, target numbers and target categories, in traffic scene images, Lower scale features mainly capture local details and texture information. Higher scale features are crucial for capturing global and semantic information of traffic scenes. The outputs of the four layers of the Res2Net network precisely represent feature information at different scales, so we adopted a pyramid structure feature fusion strategy. This strategy is prone to losing high-level features during the downsampling process. Therefore, the downsampling module integrates SE attention mechanism, which can adaptively adjust the importance of each channel and make it easier to learn advantageous important features. As shown in (B) in Fig 11, the network with AFRPM structure can better focus on the overall characteristics of the accident vehicle, such as the model paying attention to the state of front wheel and window damage, This is one of the important features for judging a wheel overturning, rather than just focusing on the damaged part.

**Table 4:** Module ablation experimental results.

Branches	Accuracy (%)	Precision (%)	Recall(%)	F1(%)
Res2	94.89	94.89	94.89	94.88
Res2+AFRPM	95.91	95.96	95.91	95.89
Res2+SCSAM	96.14	96.14	96.14	96.13
Res2+FDAM	95.45	95.47	95.45	95.44
Res2+AFRPM+SCSAM	96.59	96.61	96.59	96.58
Res2+SCSAM+FDAM	96.25	96.29	96.25	96.24
Res2+AFRPM+FDAM	95.65	95.64	95.65	95.67
Proposed	**96.88**	**96.84**	**96.88**	**96.80**

The SCSAM module integrates spatial, channel and similarity attention mechanisms. By weighting and fusing multiple dimensions of the feature map, the spatial attention mechanism can focus on the importance of different regions in the image, enabling the model to better capture key area information in traffic scene images. The channel attention mechanism can dynamically adjust the weights of different channels, enhancing the expression of important features and suppressing noise and redundant information. The similarity attention mechanism helps to extract common features of similar regions in images. The SCSAM module can enable the model to pay more attention to important features and improve its perception and discrimination of key information. This is very important for traffic scene image classification tasks, as there are complex backgrounds, objects of different scales and local details in traffic scene images. As shown in (C) in Fig 11, the heat map of the network with the addition of the SCSAM module indicates that the network has a more accurate feature recognition of vehicle damage in traffic accidents by suppressing ambient noise and redundant information. However, we also observed that although the network’s attention to the important feature of the front wheels is stronger than the baseline, it is still relatively unclear. The standard shows that this module significantly improves the performance of the model.

The table shows that the FDAM module also has a positive effect on the model’s recognition performance. The FDAM module extracts the frequency domain features of the image by converting the feature maps to the frequency domain and utilising the characteristics of the frequency domain. The application of the frequency domain feature extraction and the frequency domain attention mechanism enables the model to better exploit the frequency domain information in images, in particular to improve its sensitivity to periodic and texture information. As shown in (D) in Fig 11, the FDAM module will cause the network to focus on the low-frequency and high-frequency components in the car image. Low frequency components typically correspond to general structure and shape information, while high frequency components correspond to detail and texture information. By increasing attention to low-frequency components, the FDAM module can better capture the overall structure and shape of the entire car, rather than focusing only on damaged parts. Meanwhile, by increasing attention to high-frequency components, the FDAM module can capture more detail and texture information, further enriching the representation of the entire car. As the mesh propagates forward, the FDAM module is placed before the last layer, which can comprehensively consider global and local information and adjust in the frequency domain. Therefore, this module, combined with the multi-scale fusion and attention modules, can better exploit these fused and enhanced features. From (E), (F), (G) and (H) in Fig 11, it can be seen that the FDAM module provides filtering and attention of frequency domain importance in the multi-scale fused feature map. Although a separate FDAM module can make the model focus on more comprehensive information, it does not have good denoising and redundancy reduction. This needs to be combined with the first two modules to make the model focus more accurate. When combined with the other two modules, the FDAM module can not only accurately focus on the characteristics of the vehicle damage, but also pay attention to the state characteristics of the rollover. The table also shows that the best performance is achieved when the three modules are simultaneously integrated into the Res2Net model, resulting in an accuracy of 96.88% and an improvement of approximately 2% in various indicators compared to the baseline.

## Discussion

The paper analyzed the Traffic-Net dataset features, comprising congested traffic, sparse traffic, traffic accidents, and fire scenes. Our analysis revealed that the importance of shallow features varies across different categories in the dataset. Specifically, shallow features play a crucial role in fire and sparse traffic categories, where objects in the images are typically prominent and isolated. Shallow features capture finer details and contain more pixel-level information, allowing them to represent low-level characteristics effectively. For instance, in fire images, shallow features are better suited to express the color and shape of flames and smoke, which are essential low-level features.

Similarly, in sparse traffic images, shallow features are more capable of conveying the spaciousness, width of roads, and distribution of vehicles, which are also low-level features. In the case of congested traffic images, shallow features are instrumental in representing traffic flow and vehicle density, which are essential low-level features. Furthermore, for traffic accident images, shallow features capture pertinent information such as the vehicle’s texture, edges, and fragments.

On the other hand, sparse or congested traffic and traffic accident images require deep features encompassing more abstract semantic information, i.e., coarse-grained features. Deep features are better suited to capture high-level characteristics such as traffic shapes, congestion or sparsity levels, and vehicle collision relationships in accident images. They also enable the representation of the state and arrangement of vehicles at the accident scene, providing valuable context. Therefore, the proposed AFRPM module in this paper constructs a feature pyramid using feature layers of different scales, taking into account the scale diversity in various traffic scene categories mentioned above. Subsequently, adaptive weights are introduced to adjust the importance of different scale features, followed by feature fusion. As a result, the classification accuracy is improved by more than 0.3%.

Numerous previous studies have emphasized the significance of attention mechanisms, such as spatial channels and frequency domain, in enhancing models’ focus and feature extraction capabilities. SCSAM enhances the model’s perception of spatial and contextual information through spatial channels and similarity attention mechanisms, validating their effectiveness in classifying traffic scene images. On the other hand, FDAM specifically focuses on weighted modulation of frequency domain features. However, incorporating FDAM alone may introduce noise or interference. Nevertheless, when the AFRPM module and FDAM are integrated simultaneously, the AFRPM module acts as a filter and optimizer for features, mitigating the noise or interference introduced by FDAM and improving overall performance. Experimental results demonstrate that the combination of AFRPM and SCSAM modules further enhances the model’s accuracy, surpassing the individual contributions of each module. This result indicates that AFRPM and SCSAM exhibit complementary advantages in this task, enhancing the model’s performance in different aspects. Compared with DSMSA-Net proposed in [35], the multi-scale feature fusion strategy proposed in this paper uses a bidirectional feature pyramid to fuse features from different levels. The scale attention unit in DSMSA-Net is more dependent on learning and requires a large amount of data. Therefore, the AFRPM structure BIFPN proposed in this paper has fewer network parameters and thus has certain advantages in computational efficiency. The multi-scale strategy of the DSMSA-Net may introduce too much complexity in some simple scenarios, resulting in a relative performance degradation. The SCSAM attention mechanism in this paper integrates attention from three aspects: spatial, channel, and similarity, while DSMSA-Net only uses spatial attention units. Basically, the improved modules in this article are all based on the image properties of traffic scenes, which can better adapt to complex and ever-changing traffic scenes.

In our research, we realised that the number of model parameters is crucial for the feasibility and efficiency of practical applications. In order to maintain the lightweight of the model, we have implemented appropriate parameter control on the proposed model, ensuring that the parameter count of the model is controlled at 102.91M. Setting this number of parameters is reasonable in balancing the model capacity and computational resource consumption. Our model uses several key modules to improve the classification performance, among which the FDAM module has a parameter count of 0.52M, the AFRPM module has a parameter count of 28.23M, and the SCSAM module has a parameter count of 51.47M. The number of parameters for these modules is determined through experimentation and tuning in order to improve the performance and expressiveness of the model while maintaining a reasonable number of parameters. It is worth mentioning that the three innovative modules in this article are pluggable, and their designs are modular and interchangeable, providing flexibility for researchers and practitioners to selectively incorporate modules based on their specific needs and requirements, and to adapt the model architecture according to their specific tasks and constraints. We validate the performance of our model in traffic scene image classification tasks by conducting a comprehensive performance evaluation. We used standard evaluation metrics and based on our experimental results, our model achieved an average inference time of 20.516ms and an FPS of 48.74. This indicates that our model has high accuracy and real-time performance in processing traffic scene images.

The proposed model achieves higher classification accuracy than previous works, essential in practical applications such as traffic surveillance, intelligent transportation systems, and autonomous driving, providing more accurate scene classification results and improving system performance and decision-making capabilities. The fusion of three modules also demonstrates the importance and effectiveness of multi-module integration, and future research can further explore different combinations and structural designs of modules.

## Failure cases

In the context of the Traffic-Net dataset, which encompasses diverse categories such as traffic accidents, fires, dense traffic, and sparse traffic, there exists a certain degree of similarity between these classes. Despite the proposed model’s incorporation of multi-scale fusion and optimized attention mechanisms to reduce category misclassifications,the confusion matrix in Fig 10 reveals the persistent challenges in distinguishing between dense and sparse traffic, as well as fires and traffic accidents The subtle variations in features for dense and sparse traffic, coupled with the influence of varying camera angles on vehicle spacing and quantities, contribute to the model’s confusion. To address this issue more effectively, we recommend the integration of a rule-based solution grounded in spatial relationships. This involves explicitly defining rules for vehicle spacing, lane width, vehicle distribution, and traffic flow dynamics to enhance the model’s ability to accurately discriminate between dense and sparse traffic scenarios. However, the implementation of a spatial relationship-based solution poses challenges in terms of intricate rule design, necessitating a profound understanding of the dynamic nature of traffic scenes and the careful balance of diverse spatial relationship factors. Challenges related to parameter tuning, coping with scene complexity, and meeting real-time requirements must also be overcome. Additionally, the potential sharing of visual features between fires and traffic accidents, such as vehicles catching fire in fire scenes and resembling damaged vehicles in traffic accident scenes, can lead to misclassifications by the model. One direct approach to mitigate this issue is to augment the training dataset with an increased number of samples depicting vehicle fires, thereby intensifying the feature prominence of fire scenes. Moreover, assigning higher weights to samples of the fire category during training emphasizes the significance of the model’s learning in fire scenarios. Nevertheless, this approach necessitates a careful balance of class weights, guarding against an over-reliance on specific class features that could compromise the model’s generalization and robustness.

## Conclusions

In this paper, this study presents an improved approach based on the Res2Net network for traffic scene image classification. The proposed Adaptive Feature Refinement Pyramid Module (AFRPM) fusion network effectively addresses the challenges posed by complex backgrounds and diverse scales in critical regions of traffic scene images. The Frequency Domain Attention Module (FDAM) captures the frequency distribution patterns among different categories. At the same time, the Spatial-Channel and Similarity Attention Module (SCSAM) enhances the model’s feature perception and extraction capabilities from spatial, channel, and similarity perspectives. The experiment shows that our model has achieved an overall good classification performance on the Traffic Net data set, with an accuracy rate of 96.88%. Ablation experiments further validate each module’s contribution to improving traffic scene image classification. In future research, it is worth exploring techniques such as model pruning, quantization, and low-rank decomposition to alleviate model complexity, thereby reducing training costs and accelerating model inference. Moreover, traffic scenes commonly involve diverse data sources, including images, videos, radar, etc. Subsequent research endeavors can thus focus on exploring network architectures and strategies for effective multimodal fusion, aiming to capture comprehensive and precise feature representations.
